# Ultrasound microbubble potentiated enhancement of hyperthermia-effect in tumours

**DOI:** 10.1371/journal.pone.0226475

**Published:** 2019-12-18

**Authors:** Deepa Sharma, Anoja Giles, Amr Hashim, Jodi Yip, Yipeng Ji, Natalie Ngoc Anh Do, Juliana Sebastiani, William Tyler Tran, Golnaz Farhat, Michael Oelze, Gregory J. Czarnota

**Affiliations:** 1 Physical Sciences, Sunnybrook Research Institute, Toronto, ON, Canada; 2 Department of Radiation Oncology, Sunnybrook Health Sciences Centre, Toronto, ON, Canada; 3 Department of Radiation Oncology, University of Toronto, Toronto, ON, Canada; 4 Department of Medical Biophysics, University of Toronto, Toronto, ON, Canada; 5 Department of Electrical and Computer Engineering, University of Illinois, Urbana-Champaign, IL, United States of America; Columbia University, UNITED STATES

## Abstract

It is now well established that for tumour growth and survival, tumour vasculature is an important element. Studies have demonstrated that ultrasound-stimulated microbubble (USMB) treatment causes extensive endothelial cell death leading to tumour vascular disruption. The subsequent rapid vascular collapse translates to overall increases in tumour response to various therapies. In this study, we explored USMB involvement in the enhancement of hyperthermia (HT) treatment effects. Human prostate tumour (PC3) xenografts were grown in mice and were treated with USMB, HT, or with a combination of the two treatments. Treatment parameters consisted of ultrasound pressures of 0 to 740 kPa, the use of perfluorocarbon-filled microbubbles administered intravenously, and an HT temperature of 43°C delivered for various times (0–50 minutes). Single and multiple repeated treatments were evaluated. Tumour response was monitored 24 hours after treatments and tumour growth was monitored for up to over 30 days for a single treatment and 4 weeks for multiple treatments. Tumours exposed to USMB combined with HT exhibited enhanced cell death (*p*<0.05) and decreased vasculature (*p*<0.05) compared to untreated tumours or those treated with either USMB alone or HT alone within 24 hours. Deoxynucleotidyl transferase dUTP nick-end labeling (TUNEL) staining and cluster of differentiation 31 (CD31) staining were used to assess cell death and vascular content, respectively. Further, tumours receiving a single combined USMB and HT treatment exhibited decreased tumour volumes (*p*<0.05) compared to those receiving either treatment alone when monitored over the duration of 30 days. Additionally, tumour response monitored weekly up to 4 weeks demonstrated a reduced vascular index and tumour volume, increased fibrosis and lesser number of proliferating cells with combined treatment of USMB and HT. Thus in this study, we characterize a novel therapeutic approach that combines USMB with HT to enhance treatment responses in a prostate cancer xenograft model *in vivo*.

## Introduction

Hyperthermia (HT), also known as thermal therapy, is a method used to treat cancer by increasing the target tissue temperature up to 40–45° Celsius (C) [1]. It is considered to be an effective treatment modality for various cancers including bone, breast, prostate, liver, and lung amongst others [2]. HT has a demonstrated ability to kill cancer cells and reduce tumour volume by destroying proteins and other components within cells [3]. Studies including both biological and clinical data have revealed that HT treatment temperatures ranging from 41–45°C are effective at enhancing responses to cancer therapies including chemotherapy and radiation therapy [4] [5]. A significant reduction in the size of tumours of various types, including appendix, brain, breast, bladder, cervix, esophagus, melanoma, sarcoma and others has been reported with combined HT and radiation treatment [1] [5] [6]. Enhancement of anticancer drug effects has also been observed when combined with HT. However, a study carried out by Pelz *et al*. in patients with peritoneal carcinomatosis demonstrated that HT combined with chemotherapy (cisplatin, doxorubicin, mitomycin C, oxaliplatin) can paradoxically cause interference in cytotoxic effect by impacting the tumour microenvironment. This resulted in a protective effect, diminishing cell death, due to the activation of several heat shock proteins (HSPs), which were overexpressed in the tumours after HT treatment [7]. Hence before HT can become a standard treatment for cancer, further investigation has been required. As a result, various clinical trials have been ongoing [8].

Microbubbles (MB) have become a significant area of investigation in the field of biomedical research due to their echogenicity, making them effective contrast agents for diagnostic ultrasound. MB are micron-sized spheres composed of a gas core (such as perfluorocarbon) stabilized by a protein, lipid, or polymer shell. When stimulated by ultrasound, MB undergo a mechanical perturbation, which can induce structural changes to neighboring cell membranes [9]. Furthermore, their dynamic response to ultrasound stimulation makes them an ideal agent for the targeted delivery of drugs and gene therapy for the treatment of cancer [10]. Studies have shown significant therapeutic effects resulting from ultrasound-stimulated microbubble (USMB) treatments. McDannold *et al*. demonstrated that using ultrasound in combination with MB, resulted in the temporary disruption of the blood-brain barrier (BBB) in rabbits while causing very limited or no harmful effects on other parts of the brain. The study demonstrated the possibility of drug delivery in the brain using USMB [11]. A number of *in vitro* studies have also indicated the therapeutic effects of USMB when combined with other treatment modalities including chemotherapy, radiation, and HT. Karshafian *et al*. demonstrated that combining USMB with ionizing radiation to treat acute myeloid leukemia (AML-5) cells *in vitro* resulted in an enhancement of cell death by ~35% when compared with ultrasound alone or radiation alone [12]. The study also indicated that the enhancement effect of USMB on radiotherapy was dependent on the ultrasound pressure and MB concentration used. *In vitro* work by Ghoshal & Oelze demonstrated that a combination of USMB and HT resulted in more than 58.8% cell death compared to less than 30% and 10% cell death in cells receiving USMB only or HT only, respectively [13].

The motivation of the work proposed here comes from a large body of pre-clinical work carried by Czarnota et al. however here incorporating HT instead of radiation. That extensive past work utilized acoustically-stimulated microbubbles to enhance the effect of radiation therapy in endothelial cell models as well as in various tumour xenograft models including breast, bladder and prostate tumours [14] [15] [16] [17]. More specifically, data from experimental treatment of prostate tumour (PC3) xenografts demonstrated a 10 to 40-fold greater cell kill and significant vascular disruption with one single treatment of USMB and radiation within 24 hours. The use of ultrasound-stimulated microbubble-mediated mechanical disruption is recognized to perturb the vascular endothelial lining leading to enhanced vascular disruption. Specifically the approach sensitizes endothelial cells to radiation through ASMase-dependent ceramide-formation resulting in a supra-additive effect [14]. Studies have also indicated that exposure to HT alone is known to cause significant damage to endothelial cells and inhibits angiogenesis [18] possible through a similar mechanism. We postulate that a similar synergy may exist when ultrasound-stimulated microbubbles are used in combination with HT.

Endothelial cell proliferation and sprouting angiogenesis enable cancers to initiate and progress. Having therapeutic interventions which influence these two phenomena may improve overall tumour responses by altering the microenvironment. Cells within a tumour release vascular endothelial growth factor (VEGF), which is an essential element for survival, proliferation, and the migration of endothelial cells as well as in the regulating sprouting angiogenesis [19] [20] [21] [22]. Previous research has demonstrated that hyperthermic treatment of tumours suppresses the production of VEGF that eventually inhibits endothelial cell proliferation *in vivo* and *in vitro* [23]. Thus one may envisage that prior treatment of tumour with ultrasound-mediated microbubble will selectively sensitize the tumour tissue to HT which will lead to increased tumour response.

In the work here, the combined effect of USMB with HT in an *in vivo* prostate cancer xenograft model was investigated. Tumour responses were assessed at 24 hours and longitudinally with single treatments and multiple treatments for up to over 30 days and 4 weeks respectively. Histopathological techniques including the terminal dUTP nick-end labeling (TUNEL) for cell death, cluster of differentiation 31 (CD31) for vascular index, Masson's trichrome staining for fibrosis, and Ki-67 staining for cell viability were used to characterize tumour response. The combination of USMB with HT resulted in increased cell death, decreased vascularity and superior tumour growth inhibition when compared to USMB or HT alone for 24 hour cohort animals. Additionally, long-term data from combined USMB and HT treatment demonstrated a reduced vascular index and decreased tumour volume. Further the results indicated areas of fibrosis in addition to a reduction of proliferating cells with combined treatment.

## Materials and methods

### Cell and tissue culture

Prostate cancer cells (PC3) from the American Type Culture Collections (ATCC, Manassas, VA, USA) were maintained in RPMI1640 medium from Multicell (cat# 350–000), containing 10% FBS (Hyclone, characterized) and 1% Penicillin-Streptomycin (Gibco 15140). Cells were allowed to reach confluency while incubated at 37°C and 5% CO2. In preparation for injection, cells were washed with PBS, detached and collected using 0.05% Trypsin-EDTA (v/v) (Invitrogen, Carlsbad, USA) at room temperature. Cells were centrifuged at 200g for 10 min at 4°C and cell pellets were isolated and re-suspended in 100 μl phosphate buffered saline (PBS) per 5×10^6^ cells.

## Animals

Tumours were induced by injecting 5 × 10^6^ PC3 cells subcutaneously in the hind leg of male severe combined immuno-deficient (SCID) CB-17 mice (Charles River Inc., Wilmington, MA, USA). The tumours were allowed to grow for 3–4 weeks, at which point they reached approximately 8–10 mm in size.

All mice were anesthetized prior to treatment by an intraperitoneal injection of a mixture consisting of ketamine (100 mg/kg), xylazine (5 mg/kg) and acepromazine (1 mg/kg) (Sigma, Burlington, ON, Canada). Anesthetized mice were monitored visually and kept near heat lamps to maintain mouse body temperature.

## Ethics statement

All *in vivo* animal experiments were conducted in accordance with policies of the animal care committee at Sunnybrook Health Science Centre (Comparative Research), under animal use protocol # 18–395 and in accordance with the Canadian Council on Animal Care Guidelines. This study was approved by the animal care committee at Sunnybrook Health Science Centre (Comparative Research), at Sunnybrook Health Science Centre, Toronto, Canada. Animals were monitored and handled properly throughout the experimental procedure to minimize suffering or pain. After experiments animals were sacrificed using standard operating procedures stated by comparative research that included anesthetic overdose and cervical dislocation.

## Experimental design

In this study, peak negative ultrasound pressures of 0 kPa, 126 kPa, 246 kPa, 570 and 740 kPa were used with MB and various HT treatment durations including 0, 10, 20, 30, 40 and 50 min were tested. Experiments consisted of a short-term study lasting 24 hours, where cell death and vascular effects on tumour were assessed, and two long-term studies (over 4 weeks each), where effects on tumour growth delay were observed with repeated measurements of tumour size. For the 24 hour study, all permutations of the above parameters were tested and conditions were selected for multiple treatment experimentation. In those experiments 5 animals per group were used with 30 conditions for 150 animal’s total. In the long term studies one study used 25 animals (n = 5 per group) for evaluation of single treatments over 4 weeks.

For the long term studies, only ultrasound pressures of 0 kPa and 570 kPa and HT treatment durations of 0 min and 50 min were used. The other study here used 4 experimental treatment groups with 20 animals in each group with 5 animals sacrificed each week. This then used 20 animals treated for 1 week, 15 animals treated for 2 weeks, 10 animals treated for 3 weeks, and 5 animals treated for 1 week) for an additional 80 animals in the study. In total this works overall used 255 animals.

Definity® bubbles (perfluoropropane gas/liposome shell, Lantheus Medical Imaging, Inc., North Billerica, MA, USA) were activated by shaking using a Vialmix® (Lantheus Medical Imaging, Inc., North Billerica, MA, USA) device at 3000 rpm for 45 seconds. MB was administered to a final concentration of 1% (v/v). The MB solution was injected intravenously in mice using a 26-gauge tail vein catheter and was followed immediately by a 150 μl 0.2% heparin/saline flush. During the time of injection, mice were mounted using a custom-built mounting plate and partially submerged in a degassed water bath at 37.5°C, ensuring the tumour bearing limb was completely immersed in water.

For the ultrasound-stimulated MB procedure, an ultrasound transducer with 500 kHz central frequency (IL0509HP, Valpey Fisher Inc, MA) and an aperture diameter of 28.6 mm was used. The transducer was focused at 85 mm with a focal zone -6 dB beam-width of 31 mm. Animals received a 5 min exposure to 16 cycles tone burst at 500 kHz with a 3 kHz pulse repetition frequency and with a total ultrasound exposure time of 750 milliseconds.

## Hyperthermia treatment

Hyperthermia treatment was administered using a 43°C water bath. Mice were mounted upright in a tube with an opening at the closed end to provide an air vent and an opening in the lid for the leg and tail to pass through. The tail of the mouse was taped to the side of the tube so only the tumour-baring limb was submerged in the water. Hyperthermia exposure time ranged from 0 to 50 min.

## Histology and immunohistochemistry

Tumours were excised 24 hours after treatments, dissected in half and fixed for 2 hours in room temperature in 1% paraformaldehyde (PFA). Subsequently, they were immersed in 70% ethanol (v/v) for 48 hours at 4°C. Fixed specimens were embedded in paraffin blocks and sectioned (5 μm thick) and placed on glass slides for staining.

**Fig 1:**
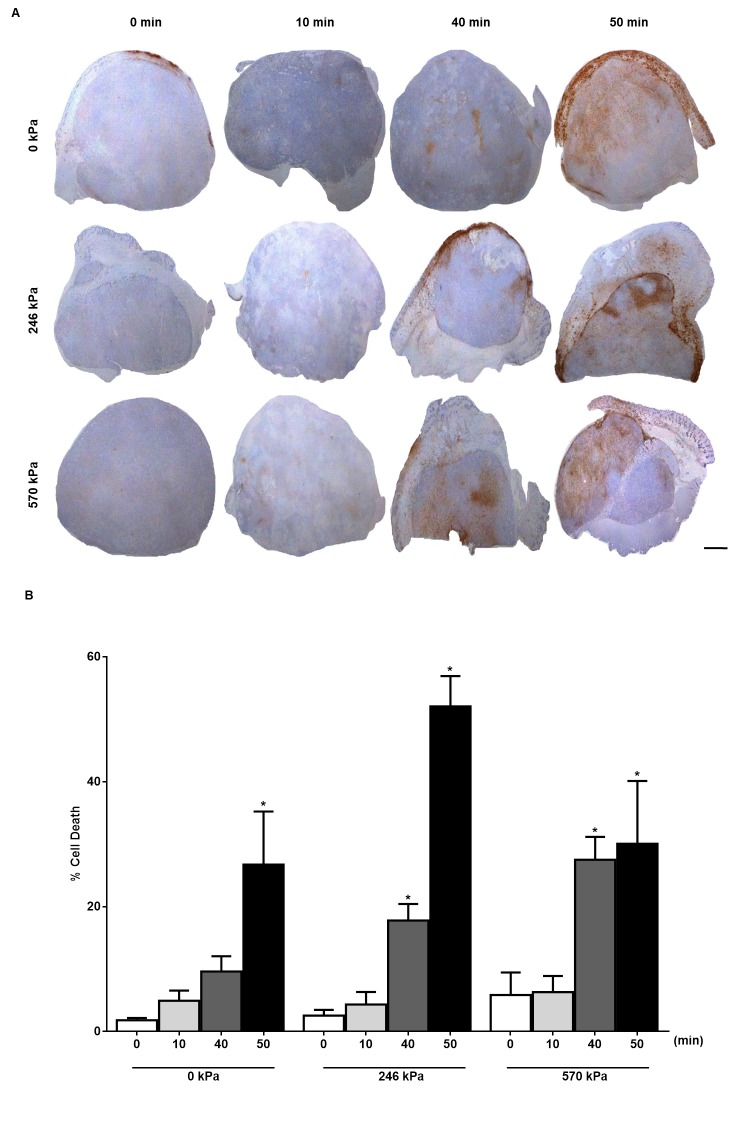
Cell death detection in PC3 xenograft tissue. (A) TUNEL stained sections of PC3 xenograft tumours treated with different conditions including USMB only, hyperthermia only and combined (USMB + Hyperthermia). Definity microbubbles were stimulated at various ultrasound pressures including 0 kPa, 246 kPa and 570 kPa (rows). Hyperthermia treatment durations were 0, 10, 40 and 50 min exposure at 43°C (columns). Scale bar: 1 mm. (B) Quantified TUNEL stained sections indicating increased cell death with increased hyperthermia treatment time and ultrasound pressure (n = 3–5). *P*-values (*p*≤0.05) are indicated by an asterisk*.

**Fig 2:**
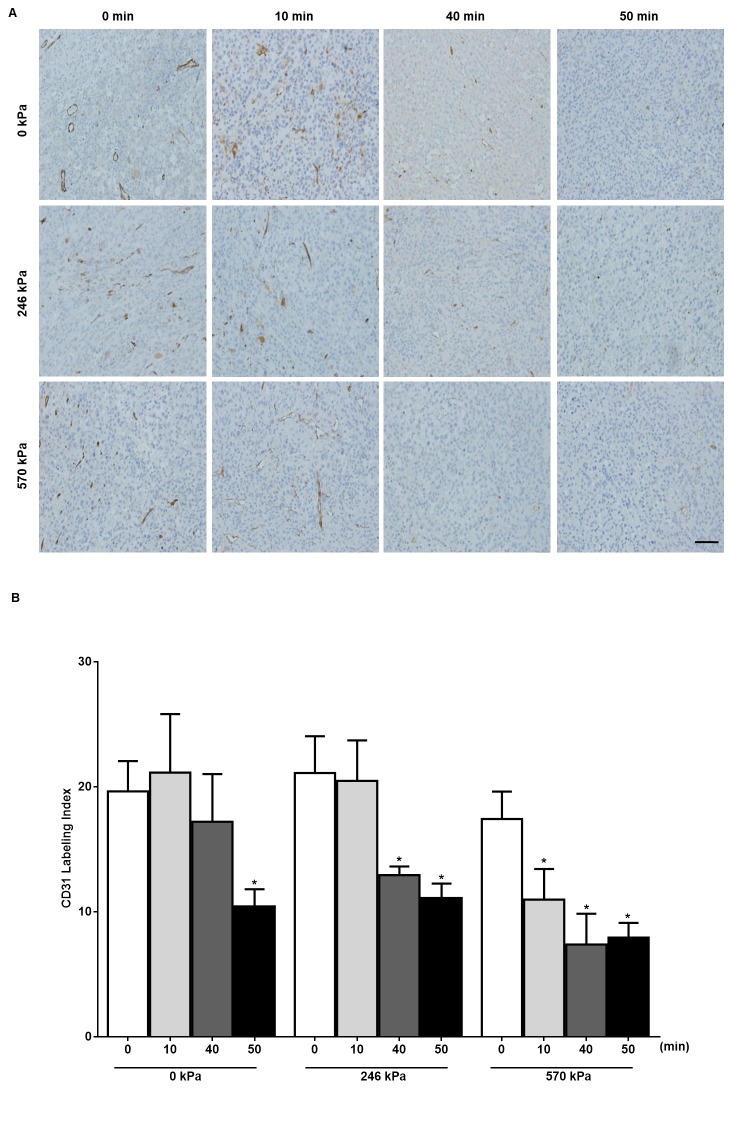
Assessment of vascular density with CD31 labeling. (A) Tumours treated with different ultrasound pressures and heating times were sectioned and stained with CD31 to detect the presence of endothelial cells in blood vessels. Scale bar: 50 μm. (B) Quantification of vascularity from whole tumour sections measured from high magnification microscopy indicates a decrease in vascularity with combined treatment (570 kPa, 40 min) when compared with heat alone 0 kPa, 40 min (n = 3–5). *P*-values (*p*≤0.05) are indicated by an asterisk*.

For the detection of apoptosis, TUNEL staining, a widely accepted in situ technique that labels nuclear DNA fragmentation was used. The stained sections were then digitized using a low-magnification light microscope (Leica MZ FL III, Leica Microsystems, Concord, ON, Canada). The cell death percentage was quantified from digitized TUNEL image files using an in-house program developed in MATLAB (Mathworks, Natick, MA, USA) (Fig 1A & 1B). For the assessment of vascularity, CD31-immunohistochemistry was used. Stained micro-vessels were counted within the entire cross-section area of the tumour slice for each tumour using Leica CD100 microscope (10x objective lens, 1MPixel Leica DC100 video camera, 2 GHz PC operating Leica IM1000 software). Vascular density was measured by selecting 10 region-of-interests (ROIs) randomly per animal and tumour. The vascular index (VI) was determined as the ratio of the sum of the intact luminal vessel to the total number of vessels. The total number of vessels includes all intact luminal vessels and ruptured vessels (Fig 2A & 2B). Additionally, Masson Trichrome staining was performed to detect fibrosis (a post cell death process) in tumours and Ki-67 staining was conducted on the tumour samples to determine the fraction of proliferating cells as a measure of cell viability. The stained sections of Masson Trichrome and Ki-67 were digitized using a high-magnification light microscope as mentioned above. From each tumour section, 5 ROIs were selected randomly.

Color-based segmentation of histologically-stained digital images was performed in order to quantify objectively the amount of fibrosis and tumour cell proliferation, associated with USMB and HT therapy. Automated analysis was performed using the roicolor method in MATLAB to select ROI based on the specified color threshold. Masson’s trichrome-stained histological images depict areas with collagen fiber deposits, resulting from fibrosis, as regions with blue/green color. The percentage of collagen was obtained by dividing the number of pixels with blue/green color by the total number of pixels in the image. Similarly, cell proliferation marker Ki-67-stained images depict the nuclear area in actively proliferating cells as a brown region. The Ki-67 labeling index was obtained by dividing the number of brown pixels with a total number of pixels in the image.

**Fig 3:**
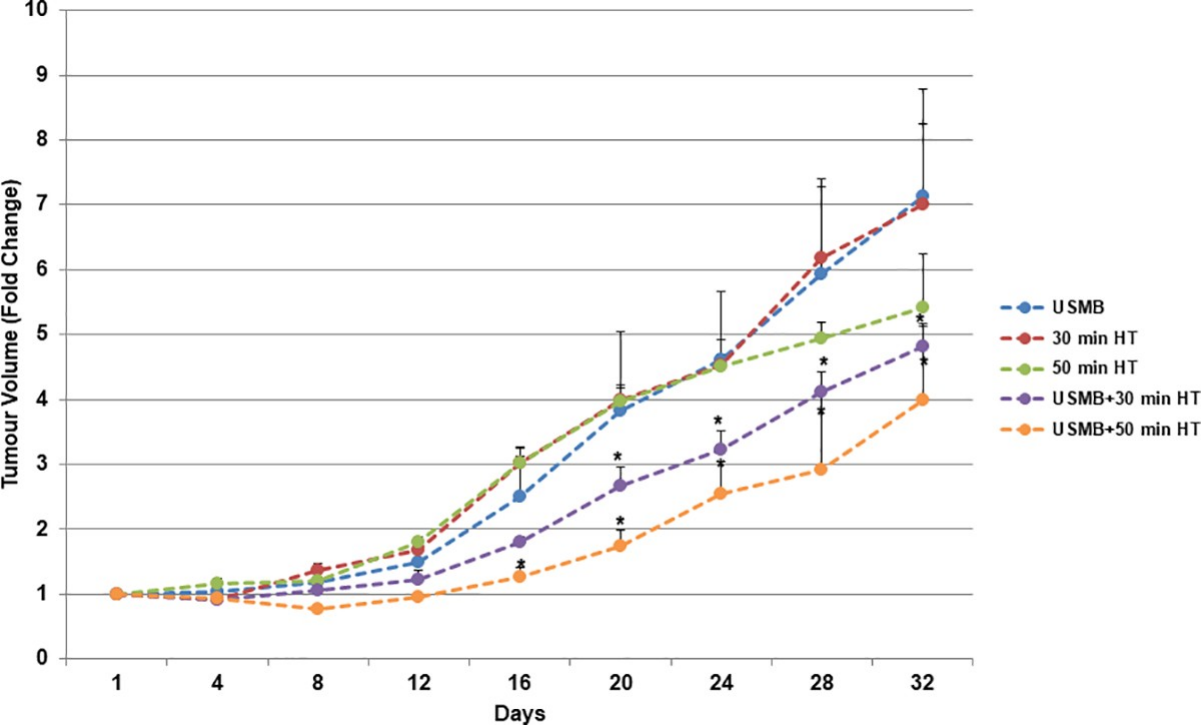
Effects of USMB and HT on tumour size and growth delay (30-day cohort): Tumour growth delay was monitored for 30 days (single treatment group). Tumour volume fold change (relative to the start day) is plotted for each treatment group (n = 4–5). *P*-values (*p*≤0.05*) represents a significant difference between the heat only and combined treatment at consecutive days.

**Fig 4:**
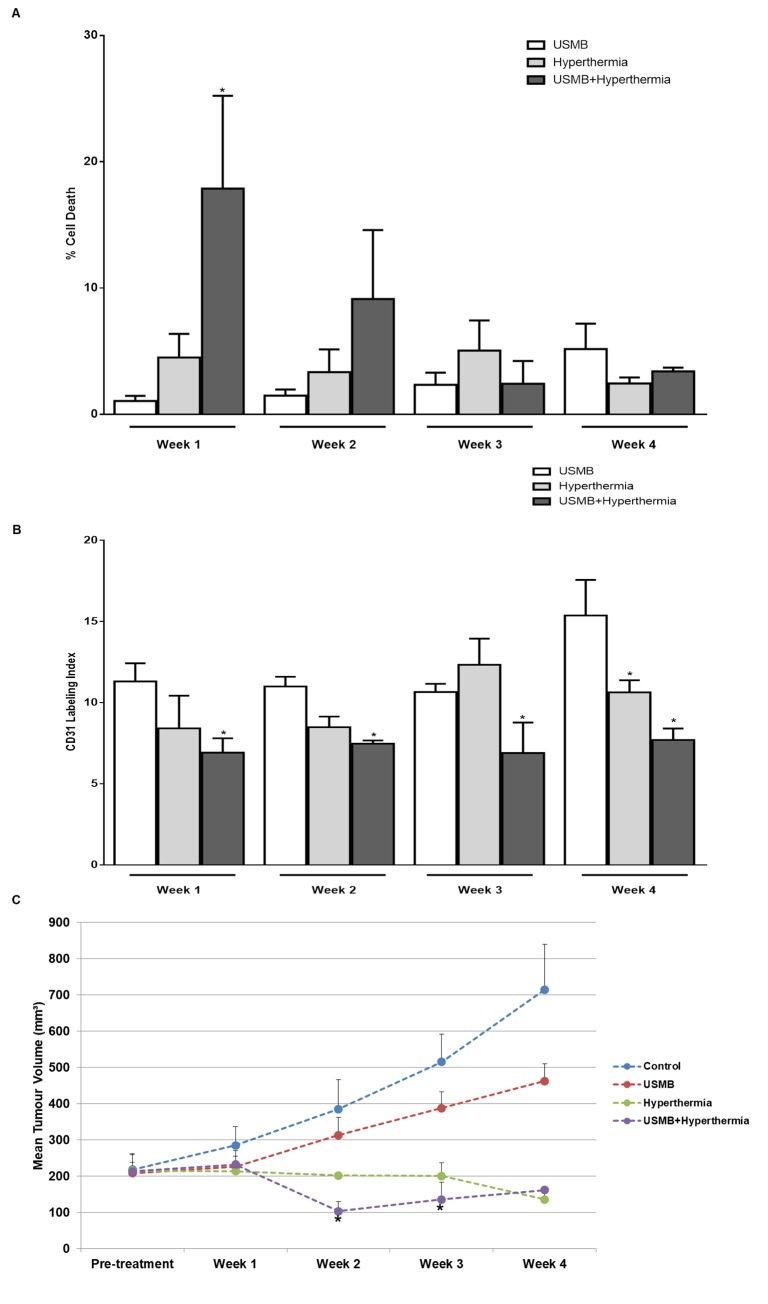
Effects of USMB and HT on cell death, vascularization and tumour size for 4 weeks cohort. (A) Cell death quantified from low magnification images of TUNEL-stained whole tumour sections. (B) Mean tumour vasculature quantified from high magnification images of CD31 stained whole tumour sections. (C) Mean tumour volume measured weekly over a period of four weeks, (n = 3–5). *P-*values (*p*≤0.05) are indicated by an asterisk* in the graph.

**Fig 5:**
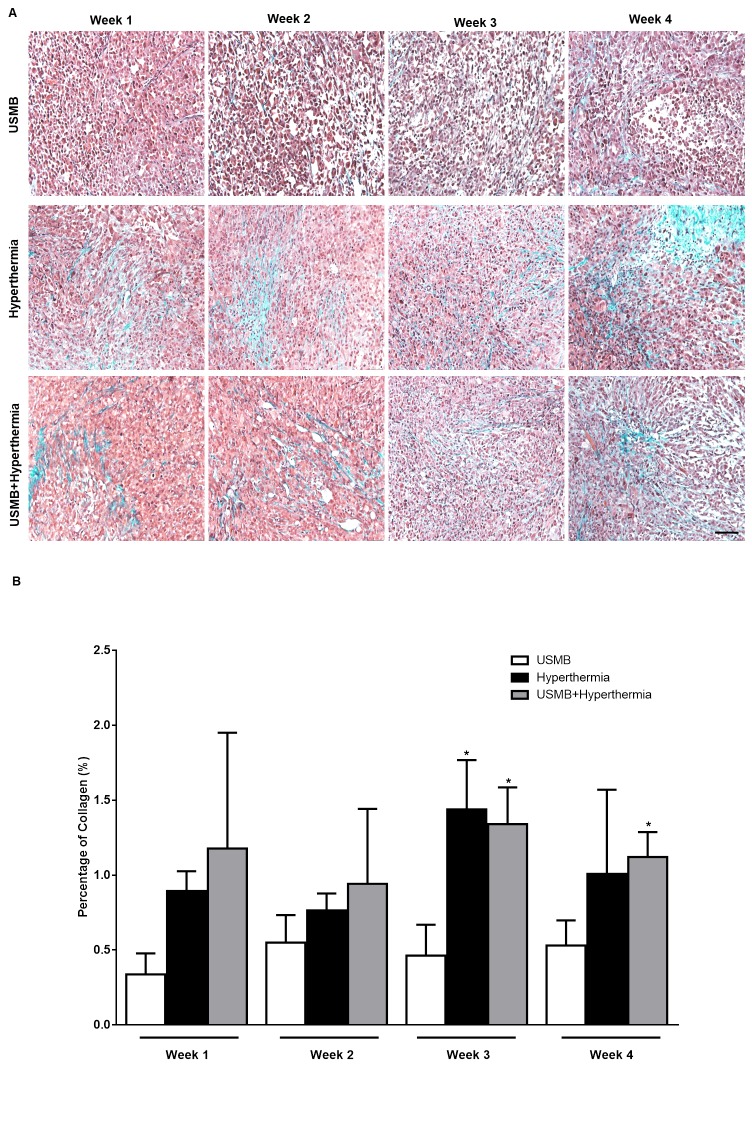
(A) High magnification representative Masson's trichrome-stained sections from tumours in each treatment group. An increase in collagen staining was observed in tissue sections (light blue color) from tumours treated with heat only and those receiving the combined treatment when compared to tumours treated with USMB alone at weeks 2, 3 and 4. Scale bar: 50 μm. (B) Quantified analyses of Masson's trichrome images, indicating an increased level of fibrosis with the hyperthermia only and combined treatments (n = 3–5). *P-*values (*p*≤0.05) are indicated by an asterisk* in the graph.

**Fig 6:**
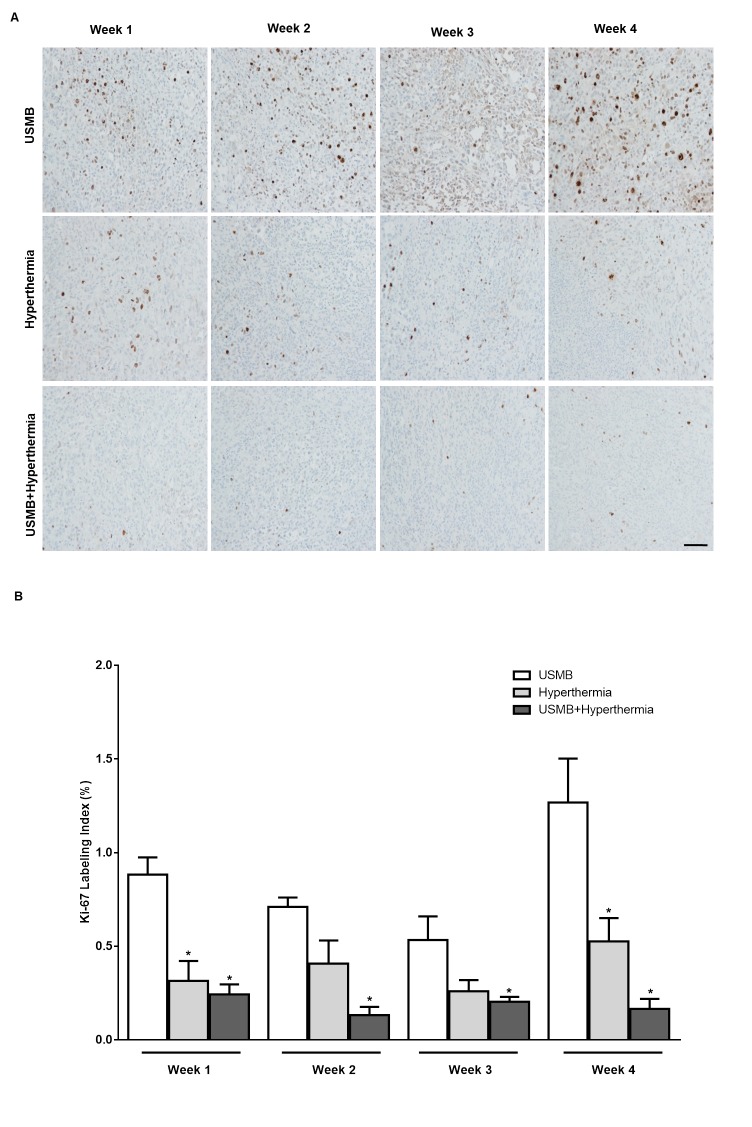
(A) Ki-67 Stained high magnification histological sections showing the growth fraction of the cell population at week 1, 2, 3 and 4 for USMB only, heat (50 min) alone and USMB + heat (50 min). Scale bar: 50 μm (B) Quantification of Ki-67 images indicates that exposure of xenografted tumors to combined treatment of USMB and hyperthermia resulted in a larger decrease in Ki-67 labeling indices compared to USMB alone or hyperthermia only (n = 3–5). *P*-values (*p*≤0.05) are indicated by an asterisk* in the graph.

Longitudinal studies were performed in order to verify the efficacy of multiple combined treatments compared to either USMB or HT alone and were composed of two experimental cohorts. The first was conducted over 4 weeks at the start of which each animal received a single treatment consisting of either USMB only, HT only, or a combined treatment consisting of USMB + HT (30 min) or USMB + HT (50 min) (Fig 3). A second cohort received multiple treatments consisting of two treatments per week over a period of 4 weeks (Fig 4A, 4B & 4C) (Fig 5A & 5B) (Fig 6A & 6B). In that group, treatments were administered on days 1 and 5 and consisted of either USMB only, HT only or a combined treatment of USMB + HT (50 min).

For the multiple treatment studies (Fig 4), only one combined treatment using USMB + HT (50 min) for maximal effect was administered. Tumour cell death (Fig 4A) and tumour vascular density (Fig 4B) were assessed by immunolabeling using TUNEL and CD31 stains, respectively. In addition, tumour size was measured weekly up to 4 weeks (Fig 4C). Further treatment effects on biological activity were assessed using Masson's Trichrome staining to delineate fibrosis and Ki-67 for cell proliferation (Fig 5A & 5B) (Fig 6A & 6B). Data were also collected for animals that remained untreated (control) (S3 Fig).

### Tumour growth

Tumour growth was assessed for long term cohort studies, which lasted for over 4 weeks for the single treatment group and a similar time frame for the multiple treatment groups. Tumour size was measured using digital calipers every 2–3 days and weekly in order to determine the length (L) and width (W). Tumour volume was determined by using the modified ellipsoidal formula (volume = length × width^2^/2). The change in volume for each tumour measurement was calculated relative to initial tumour volume for over 30 day-study and the means and standard errors were plotted for each treatment for 4 weeks study.

## Statistical analysis

Statistical analysis was conducted using Graph Pad/InStat 3.0). Statistical significance was determined by analysis of variance (ANOVA) followed by a Bonferroni’s selected comparisons test. *P-*values (*p*≤0.05) were regarded as statistically significant and are indicated by asterisk*.

## Results

This work aimed to study the acute (after 24 hours) and long-term effects (at 4 weeks) of USMB and HT in a mouse model of prostate cancer (PC3). For the 24 hour cohort, animals were submitted to treatment with USMB consisting of 1% (v/v) Definity MB stimulated at various ultrasound pressures of 0, 126, 246, 570 and 740 kPa followed with heat treatment, 43°C for 0, 10, 20, 30, 40, and 50 min. A total of 30 different treatment conditions were investigated and a total of 150 animals (5 animals per condition) were included for the 24 hour cohort. For the 4 week cohort, animals were divided into five groups consisting of USMB (n = 4), 30 min HT (n = 5), USMB+ 30 min HT (n = 5), 50 min HT (n = 5), and USMB+ 50 min HT (n = 5). For this cohort, a total of 4–5 animals per week were included for each treatment group including control, USMB, 50 min HT, and USMB+50 min. Untreated mice were labeled as control (0 kPa+ 0 min).

### The combination of USMB and HT increases cell death in PC3 tumours *in vivo*

Results indicated an increase in cell death with increasing HT treatment duration. Cell death, confirmed by the presence of dark brown regions in TUNEL stained tumour sections (Fig 1A) increased with HT time and with exposure to USMB. The 50 min, heat only (0 kPa) treatment resulted in a significant amount of cell death (26.73 ± 8.51%, mean ± SE) (*p*≤0.05) when compared to the untreated control (1.80 ± 0.37%), 10 min heat only (4.93 ± 1.62%) and 40 min heat only (9.64 ± 2.43%) treatments (Fig 1B). Similarly, USMB treatment at 246 kPa, combined with 40 min (17.80 ± 2.62%) (*p*≤0.05) and 50 min (52.08 ± 4.82%) (*p*≤0.05) heat caused a significant increase in cell death compared with the same USMB treatment combined with 0 min (2.55 ± 0.92%), or 10 min (4.32 ± 1.99%) of heat. Furthermore, USMB treatments at 570 kPa combined with 40 min of heat exhibited a significantly higher amount of cell death (27.50 ± 3.66%) (*p*≤0.05) compared to 0 min (5.84 ± 3.63%) and 10 min (6.30 ± 2.60%) of heat. At the same pressure, 50 min heat did not cause any further drastic increases in cell death (30.07 ± 10.07%) compared to 40 min (27.50 ± 3.66%), however the cell death index was significantly higher than what was observed at 0 min (5.84 ± 3.63%) and 10 min (6.30 ± 2.60%) of heat. Comparisons were also made to verify the differences in cell death between the different treatment conditions. Fifty minutes of heat produced a significant level of cell death regardless of ultrasound pressure, indicating that this duration of heat application is not enhanced by the USMB treatment. A 40 min heat treatment, however, is significantly enhanced (*p*<0.05) by USMB at a pressure of 570 kPa.

Interestingly, 30 min of heat combined with 126 kPa indicated significant cell death (S1 Fig) however with other USMB treatments, shorter durations of heating (20 and 30 min) were not enhanced in terms of cell death. High pressures (740 kPa) did not change much in terms of cell death produced compared to 570 kPa.

### USMB and HT cause an additive effect resulting in decreased vasculature in PC3 tumours *in vivo*

The disruption of blood vessels resulting from USMB and HT treatments was investigated using CD31 immunohistochemistry (Fig 2A). In the heat only treatment group, vascular labeling was significantly lower with 50 min (VI = 10.43 ± 1.369, mean ± SE) (*p*≤0.05) of heat compared to the untreated control (VI = 19.63 ± 2.42). In the USMB treated tumours, at 246 kPa, a significant reduction in vascular labeling was observed with 40 min (VI = 12.93± 0.6960) (*p*≤0.05) and 50 min (VI = 11.10 ± 1.156) (*p*≤0.05) compared with 0 min (VI = 21 ± 3). At 570 kPa, 40 min (VI = 7.380± 2.460) (*p*≤0.05) and 50 min (VI = 7.933± 1.184) (*p*≤0.05) of heat reduced the vascular labeling significantly when compared to 0 min (VI = 17.43 ± 2.185). When comparing between the different ultrasound pressures, the combined treatment at 570 kPa with 40 min of heat indicated decreased vascularity (VI = 7.380± 2.460) (*p*≤0.05), which was significant compared to 40 min of heat alone (VI = 17.20 ± 3.811). Further increases in the heating time did not change the effect on the vasculature while heating times of 20 and 30 min had effects intermediate to 10 and 40 min. The lower and higher pressures used, 126 kPa, and 740 kPa showed a similar trend of reduction in vascular labeling as 570 kPa (S2 Fig).

### Ultrasound-activated microbubble treatment combined with hyperthermia results in inhibition of PC3 tumour growth

In the single treatment over 30 day-study (Fig 3), tumour volume fold change was determined for each treatment conditions (relative to initial tumour volume). Results from the first combined group, receiving USMB + HT (30 min) resulted in a delay of tumour growth. At day 20 these animals exhibited tumour volume fold change of (2.67 ± 0.29, mean ± SE) (*p*≤0.05) compared to the group receiving 30 min of HT only with a fold change of (3.98 ± 0.19). Similarly, this persisted to day 24, 28 and 32 with a fold change of (3.21 ± 0.29) (*p*≤0.05), (4.12 ± 0.30) (*p*≤0.05) and (4.81 ± 0.35) (*p*≤0.05) respectively compared to 30 min of HT only with a fold change of (4.52 ± 0.40) at day 24, (6.18 ± 1.09) at day 28 and (7.00 ± 1.24) at day 32. Within the group receiving the combined treatment of USMB + HT (50 min), tumour growth was delayed longer, for example at day 16, tumour volume fold change was found to be (1.26 ± 0.14) (*p*≤0.05), when compared to the group receiving 50 min of HT only with a fold change of (3.02 ± 0.23). Tumours grew more slowly after the combined treatment USMB + HT (50 min) with a fold change at day 20 (1.73 ± 0.26) (*p*≤0.05), day 24 (2.53 ± 0.50) (*p*≤0.05), day 28 (2.92 ± 1.19) (*p*≤0.05) and day 32 (4.00 ± 1.12) (*p*≤0.05) compared to HT (50 min) alone with a fold change of (3.96 ± 0.24), (4.50 ± 0.19), (4.93 ± 0.24) and (5.41 ± 0.83) at day 20, 24, 28 and 32 respectively.

### Changes in histopathology and tumour size over the course of multiple USMB and hyperthermia treatments

Quantification of TUNEL staining indicated tumours receiving USMB only treatment demonstrated no appreciable increase in areas of cell death at weeks 1, 2, 3 and 4. Further in the heat (50 min) only treated tumours, negligible area of cell death was visible at weeks 1, 2, 3 and 4. Tumours receiving the combined treatment of USMB + heat (50 min) exhibited large areas of cell death at week 1, however, this was less with subsequent treatments. Specifically, after one week of treatment, a significant increase in cell death was observed in tumours receiving the combined treatment of USMB + HT (50 min) (17.87 ± 7.36%) (*p*≤0.05) compared to those receiving USMB only (1.06 ± 0.40%) or HT (50 min) only (4.50 ± 1.88%).

Results from the quantification of CD31 staining revealed that vascular labeling was significantly reduced in tumours receiving the combined treatment USMB + HT (50 min) at week 1 (VI = 6.92 ± 0.87) (*p*≤0.05), week 2 (VI = 7.47 ± 0.19) (*p*≤0.05), week 3 (VI = 6.90 ± 1.88) (*p*≤0.05) and week 4 (VI = 7.70 ± 0.70) (*p*≤0.05) compared to USMB only indicating vascular labeling at week 1 (VI = 11.31 ± 1.12), week 2 (VI = 11 ± 0.59), week 3 (VI = 10.65 ± 0.50) and week 4 (VI = 15.38 ± 2.19). Differences compared to HT alone were less (Fig 4B).

Tumour size after multiple treatments experiments was assessed up to 4 weeks after treatment (Fig 4C). Mean tumour volume was calculated for each treatment conditions including control (no treatment), USMB only, HT (50 min) only and combined treatment USMB + HT (50 min). Tumour receiving no treatment or USMB only group grew rapidly during the course of the experiment with tumour volume reaching (714.0 ± 125.5 mm^3^, mean ± SE) and (462.3 ± 47.52 mm^3^) at week 4 compared to pre-treatment (217.6 ± 42.07 mm^3^) and (208.1 ± 9.74 mm^3^) respectively. However, in the case of HT alone, no significant changes in tumour volume were noticed at week 1, 2 and 3. At week 4 reduction in tumour volume was observed but it was not statistically significant compared to pre-treatment. Combination treatment USMB+ HT (50 min) resulted in substantial tumour volume reduction with (103.0 ± 27.16 mm^3^) (*p*≤0.05) at week 2, (135.5 ± 46.99 mm^3^) (*p*≤0.05) at week 3 and (161.2 ± 6.123 mm^3^) at week 4 compared to pre-treatment group (212.8 ± 49.05 mm^3^).

Specifically, since tumours receiving the combined treatment did not indicate any significant cell death, Masson's trichrome staining of tumour sections was performed to test whether the cells converted viable tissue to fibrosis resulting in collagen deposition. Fibrosis was characterized by staining for the accumulation of abnormal extracellular matrix components resulting in enormous tissue repair. Trichrome staining is mostly used to detect the presence of collagen [24]. Trichrome staining indicated negligible deposition of collagen (light blue areas) at week 1, 2, 3 and 4 in tumours receiving USMB only and prominent deposition of collagen at weeks 2, 3 and 4 in tumours receiving heat (50 min) only. Similarly, in tumours receiving the combined treatment, significant collagen staining was observed at weeks 3 and 4 (Fig 5A & 5B). Quantification of Trichrome staining revealed a significant amount of increase fibrotic tissue followed combined treatment USMB + HT (50 min).

In order to better characterize treatment effects on cell activity, Ki-67 immunostaining was conducted to analyze the fraction of tumour cell proliferation and growth for further investigation of multiple treatment effects on tumour regression (Fig 6A & 6B). Treatment with USMB only resulted in tumour with a Ki-67 index of (0.88 ± 0.09%, mean ± SE) at week 1, (0.71 ± 0.04%) at week 2, (0.53 ± 0.12%) at week 3 and (1.26 ± 0.23%) at week 4. Tumours treated with HT alone yielded an index of (0.31 ± 0.10%) at week 1, (0.40 ± 0.12%) at week 2, (0.26 ± 0.05%) at week 3 and (0.52 ± 0.12%) at week 4. Tumours treated with combined USMB + HT (50 min) showed a further decrease in Ki-67 labeling indices to (0.24 ± 0.05%) (*p*≤0.05) at week 1, (0.13 ± 0.04%) (*p*≤0.05) at week 2, (0.20 ± 0.02%) (*p*≤0.05) at week 3 and (0.16 ± 0.05%) (*p*≤0.05) at week 4 compared to USMB. A significant reduction in Ki-67 index was observed at week 4 when compared between HT alone and USMB + HT (50 min).

## Discussion

Several studies have demonstrated that ultrasound-stimulated microbubble (USMB) treatment can cause the mechanical disruption of tumour vasculature resulting in enhanced cell death [25] [14] [15] [26] [17]. A study by Al-Mahrouki et al. demonstrated that combining USMB therapy with radiation synergistically increases tumour cell death initiated from the endothelium and vascular damage. The radiation dose of a 2 Gy or 8 Gy in combination with USMB caused higher amounts of tumour cell death and vascular disruption when compared to either of the treatments alone in a prostate xenograft model. Those results revealed that combined treatments caused vascular damage with increased vessel leakiness and increased cell death [16]. The findings further concluded that the phenomena of cell death and vascular damage were dependent on a ceramide-related cell death signaling pathway [14] [27]. Similarly, combining USMB treatment with chemotherapy has also been demonstrated to enhance tumour response. In work conducted by Goertz et al. and Todorova et al. results indicated an increase in the antitumor activity of docetaxel and metronomic cyclophosphamide when combined with USMB in PC3 and MDA-MB-231 tumour xenograft model respectively. In both the studies, USMB alone or combined with a chemotherapy-induced significant reduction in tumour blood flow and cell death within 24 hours. Furthermore, within four to six weeks of the combined treatment, tumour size reduction and growth inhibition were observed [28] [29]. These studies correlated tumour growth inhibition as an effect of vascular shutdown. This supports the concept that the combination of antivascular USMB effects with cancer therapies is an effective way to target tumour vasculature.

Despite the wide use and effectiveness of radiotherapy and chemotherapy to sensitize tumours, surviving tumour cells often acquire resistance towards these therapies [30] [31]. Moreover, multiple survival pathways are also activated that allows cells to proliferate, survive, invade and circulate through the body resulting in secondary, or metastatic, tumours [30] [32]. The most commonly activated survival pathways in radio- and chemo-resistant tumours are the angiogenic signaling pathways that involve the formation of new blood vessels in a highly regulated and complex way leading to therapeutic outcome failures [33] [34]. However, over the years, there have been abundant data demonstrating that hyperthermia (HT) hinders the angiogenesis process [29]. Work has suggested that HT inhibits the production of tumour-derived vascular endothelial growth factor that suppresses endothelial-cell proliferation, and migration, and finally interferes with new vascular lumen formation [23].

Thus in this study, the rationale for combining USMB with HT is to enhance the efficacy of both modalities by targeting tumour vascular endothelium contributing overall to tumour response. Tumours were subjected to USMB treatment preceding HT by 5 hours in order to maximize responses based on previous work done with USMB. The results indicated that when combined with USMB, HT treatment resulted in cell death, which increased with HT treatment duration (heating times of 0, 10, 20, 30, 40, and 50 min were tested, 20 and 30 min added in S1 Fig). Cell death was measured by quantifying TUNEL staining of tumour sections. Decreased vascularity was observed in tumours receiving combined treatment and results were confirmed using CD31-staining, a marker to identify the presence of vascular endothelial cells.

It must be noted that in the study here at 24 hours after treatment, combined treatment with an ultrasound pressure of 570 kPa, and 40 min of heat significantly enhanced cell death and reduced the vascular index compared to treatment with ultrasound pressure of 0 kPa and 40 min of heat. Longer heating times resulted in smaller differences. However, when combined with USMB, 40 min of heating was sufficient to induce significant tumour effects. Cell death induced upon USMB is known to be pressure-dependent as shown in a study by Kim et al. [35] however in our study 246 kPa combined with 50 min heat demonstrated the highest level of cell death instead of 570 kPa or 740 kPa with same heating time. It is worth noting that the lowest ultrasound pressure of 126 kPa when combined with 30 min heat also demonstrated a significant amount of cell death (S1 Fig). It is possible that at lower USMB pressures there might be enhanced endothelial damage inducing apoptosis however as the pressure is increased the generation of oxidative stress and activation of heat-shock proteins (HSPs) may cause more thermally related cell death leading to more necrosis [36]. Data from CD31 staining revealed greater decreases in the vascular index with increases in ultrasound pressure. When using low ultrasound pressure of 126 kPa, 50 min of heat was required to achieve a significant reduction in vascularity however when a higher ultrasound peak negative pressure of 740 kPa was used, 30 min of heat was sufficient for significant vascular inhibition. Even though higher pressures resulted in significant vascular damage, time dependent vascular damage was not prominent in our study. The results indicate that cell death induced upon USMB and HT were largely the same over the ultrasound pressures tested and with the HT durations tested. Changes in the vasculature however appeared dependent on ultrasound pressure but largely invariant over the heating times studied. Several studies have confirmed time-dependent cellular effect using various *in vivo* and *in vitro* models but these have made use of USMB to perturb the vasculature in advance [37] [38].

A unifying mechanism for cell death and vascular damage through USMB is distinct from classical research in HT. Studies have shown that cell death and vascular disruption-induced upon USMB is dependent on the concentration of microbubbles as well as the ultrasound pressure applied to stimulate the microbubbles [35]. Also, the level of cell death and vascular damage is known to be directly correlated with the activation of acid sphingomyelinase (ASMase) pathway leading to ceramide generation [16]. Moreover, vascular endothelial cell apoptosis is known to be a major driver for this pathway [39] [40] [41]. On the other hand, HT-induced cellular response is mainly dependent on the aggregation, denaturation, and unfolding of proteins [42]. Both *in vitro* and *in vivo* studies have demonstrated that heat-induced apoptosis is mainly a result of pro-apoptotic proteins upregulation and activation with the involvement of reactive oxygen species (ROS) production [43]. Interestingly, HT range between 39–45°C has demonstrated enhanced blood flow followed by improved tissue oxygenation causing direct or indirect cell death leading vascular deterioration [44] [45]. The mode of cell death induced by HT is known to be time-and temperature-dependent [46] [37] [47]. Usually, temperature up to 47°C results in apoptotic cell death while increasing the temperature above 50°C exhibits necrosis as a result of tumour cells destruction [36]. Cells when treated with ≤ 43°C HT initiate the synthesis of HSP as a result of thermotolerance effect but yet not reaching the level of tumour cell cytotoxicity which mediates apoptosis. On the other hand, HSP released at a higher temperature from dead/necrotic tumour cells activates antitumor immunity resulting in tumour regression and metastasis [48]. However, recent work in HT and ceramide production has indicated effects of HT on ceramide production [49]

In the work here, USMB alone did not result in any significant effect on cell death and vascular damage, however exposure to HT only or combined with USMB revealed enhanced cellular response which suggests that advance treatment with USMB potentiates the effects of HT. Thus at this point, it is unsure if the modulation of cell death and vascular damage observed in this study is either dependent on ceramide generation or the activation of various cell death-related proteins or a combination of both.

The TUNEL and CD31 results from the 24 hour study here, which indicated increased cell death and decreased vascularity, respectively, were further validated by measuring tumour size in longitudinal studies. In the single treatment case (a tumour treated only once), tumour volume measurements conducted over a period of 30 days indicated an initial reduction of tumour volume followed by slower tumour growth in the groups receiving the combined treatment of USMB + HT (30 min) or USMB + HT (50 min). Since the 24-hour study indicated that 40 min of heat combined with USMB resulted in significantly more cell death and vascular disruption than 40 min of heat alone, heating durations of 30 and 50 minutes were selected for further investigation in the longitudinal study.

The tumour growth inhibition that followed USMB treatments has been frequently correlated with the shutdown of tumour blood flow [29] [28] [27] [50]. On the other hand, inhibition of tumour growth with HT treatments has been associated with apoptosis-linked proteins. Prior research conducted with a murine model of malignant melanoma exposure to 1 hour of HT treatment at 43°C and 45°C resulted in average tumour size reduction by 33% and 67% respectively compared to control group at 37°C. That study correlated changes in tumour size with an expression of protein levels that stimulate apoptotic pathways. Data indicated HT-treated groups expressed increased levels of active caspase-3 and phospho-H2A.X (Ser139) and reduced levels of COX-2 compared to the control group [51]. Similarly, exposure of cells and tumours with USMB *in vivo* and *in vitro* have demonstrated enhanced tumour inhibition via regulation of Bax and Bcl-2 [52] along with increased expression of cell-death related caspase-3, cleaved caspase-3, and caspase-8 [53]. Here, in our study combined USMB and HT group demonstrated greater tumour size inhibition compared to either of the group alone likely linked to the increase vascular disruption evident in the combined therapy group.

In a second longitudinal study as part of the work here, PC3 tumours received multiple treatments, two days a week over a four-week period. Results demonstrated a significant increase in cell death with the combined USMB + HT (50 min) treatment at week 1, but no significant difference in cell death was noticed followed week 2 (Fig 4A). Nevertheless, the combined treatment induced a significant decrease in vascularity at weeks 1, 2, 3 and 4 when compared with USMB only or HT (50 min) only (Fig 4B). Additionally, animals treated with USMB + HT (50 min) demonstrated significantly attenuated tumour volume at weeks 2, 3 and 4 (Fig 4C).

Previously, Goetz and group studied the longitudinal effects (over a duration of 5 weeks) of USMB treatment on the size of PC3 tumours implanted in athymic mice and demonstrated that USMB treatment alone did not impact the size of tumours significantly. However, combining USMB with the chemotherapy drug docetaxel resulted in significant tumour size reduction. The change in tumour size was interpreted being a result of vascular shutdown [28]. Similar results were found in the work here indicating no changes in tumour volume with the USMB treated group compared to the control group over 4 weeks. In contrast, the combination of USMB and HT demonstrated tumour growth inhibition starting at week 1 persisting to week 3. The precise mechanisms of USMB and HT inhibiting tumour growth remain uncertain at present however there might be several mechanisms at work. The first is that the vascular damage observed acutely is associated with observed long term tumour growth inhibition. Secondly, the activation of several apoptotic processed due to combined USMB and HT might lead to growth retardation of tumours.

In order to further study these biological responses to treatment were further assessed using Masson's trichrome staining and Ki-67 staining. The results here from Masson's trichrome staining demonstrated that in tumours treated with USMB only there was a little or no collagen deposition at weeks 1, 2, 3 and 4. In the HT (50 min) only treatment group, the presence of collagen was visible as early as week 2 and continued into weeks 3 and 4 (but only statistically significant at week 3). Similarly, the combined treatment USMB + HT (50 min) exhibited a clear increase in collagen staining in weeks 2, 3 and 4. These results indicate that treating tumours multiple times with HT either alone or in combination with USMB causes tumour cells to undergo fibrosis resulting in collagen accumulation (Fig 5A & 5B). This would likely explain why cell death was not detected readily at weeks 2, 3 and 4 in the combined treatment group since the affected cells may have already been replaced by collagen and fibrotic tissues. The results here suggest the occurrence of early phase cell death following HT only or combined treatment later followed by fibrosis formation.

The Ki-67 immunohistological assessment of tumours from combined USMB + HT (50 min) treatment group demonstrated the lowest level of actively dividing cells compared to USMB only treated group or HT (50 min) only treated animals (Fig 6A & 6B). Hence, we anticipate that exposing tumour xenograft to HT (50 min) only or combined treatment of USMB + HT (50 min) over a period of four weeks destroys the cells within a certain area. The dead cells are replaced by scar tissue which is confirmed by trichrome positive staining. However, in HT (50 min) only treated group a certain population of cells keeps dividing having the potential to form new cells as detected by Ki-67 staining. On the other hand, with combined treatment USMB + HT (50 min) there is greater cell kill with no ability to proliferate further. Overall, we can confirm that even though no changes in cell death over a period of four weeks was observed between these two groups, changes at the cellular level were detected with differences in the percentages of proliferating cells, as the combined group had significantly less Ki-67-positive cells than the other groups.

The study here investigated the new combination of USMB and HT in PC3 tumours in a mouse xenograft model. While using HT adjuvant with other cancer treatment the sequence and time interval between the two modalities is known to have a profound effect on tumour response [54] [55]. There has been ongoing discussion whether HT should be administered prior, after, or simultaneously with other treatments. Work has been conducted to explore the interaction between HT and irradiation using two different cell lines: HA-1 and EMT-6. In HA-1 cells, heating after irradiation was more effective whereas in EMT-6 prior heating demonstrated greater sensitization [56]. Several *in vivo* and *in vitro* studies have suggested that simultaneous application of heat with other cancer treatments is known to have superior tumour response because of maximum thermal enhancement achieved by reducing the interval between two treatments [55]. It remains unknown here if reversing the sequence of treatment here with the combined treatments would alter tumour responses. However, we anticipate, heating tumours before USMB might cause alteration of blood flow and tissue oxygenation that might increase the efficacy of USMB. In addition, exposing tumours multiple times with HT might initiate vascular thermotolerance leading to vessel normalization that alleviates tumour oxygen levels [57] and hence this may improve the ability of USMB to kill cancer cells. Alternatively, inducing vascular disruption in advance through a ceramide related process may prime tumors for ceramide related affects that hyperthermia may elicit [49].

The primary finding of this study is that the combination of USMB and HT caused acute and long-term effects on prostate xenograft model. Significant effects in cellular response measured by cell death and vascular damage were noted within 24 hours followed by tumour growth inhibition. Additionally, 4 weeks of USMB and HT exposure showed no measurable apoptotic cell death which may have been a result of fibrosis due to multiple treatments indicating vascular disruption. Furthermore, the combined treated group demonstrated a significant reduction in tumour volume which was associated with a relatively low percentage of proliferating cells. It is likely that the long-term tumour response observed in this study might be dependent on the vascular damage noted in the acute study.

## Conclusion and limitations

There is abundant evidence demonstrating the potential of using ultrasound and microbubbles to enhance tumour response. In this study, we investigated ultrasound-stimulated microbubbles (USMB) to enhance the effects of hyperthermia (HT). Tumours treated with the combination of USMB and HT revealed increased cell death, reduced vascularity, and superior inhibition in tumour growth and tumour cell proliferative activity when compared with either treatment alone or untreated group. The enhanced tumour response demonstrated in this study provides a basic understanding of how tumours *in vivo* respond to the combination of USMB and HT. A limitation of the work here involves addressing any tissue heating heterogeneity which can be carried out using more sophisticated hyperthermia methods. Hence the absorbed thermal dose and any non-thermal effects remain undetermined. Modern HT techniques for example modulated electro-hyperthermia technology, and MRI-guided hyperthermia using focused ultrasound enables several technical adaptation to tissue heterogeneity and can records the total absorbed dose with the ability to control the selective deposition of energy by the tumour tissue [58]. In addition, overheating may induce necrosis through oncosis rather than apoptosis during the heating process, Therefore, a technique that allows selective heating of malignant tumour cell membranes might be useful in stimulating several signaling pathways resulting in programmed cell death rather than necrotic cell death [59].

Furthermore for this technique to be successfully implemented with clinical utility further preclinical research should be conducted that includes testing this technique in large animals and or an orthotropic tumour model. In addition, the optimal time interval between HT and USMB should be investigated and the research should provide a more definitive answer to whether HT treatment should be given sequentially or simultaneously with USMB.

## Supporting information

S1 ARRIVE checklist (DOCX)

S1 Fig TUNEL cell death detection in tissue sections. Quantified cell death staining at 24 hours following treatment of PC3 xenografts with varying ultrasound pressure and hyperthermia duration: (A) 0 kPa 20 and 30 min; (B) 246 kPa 20 and 30 min; (C) 570 kPa 20 and 30 min (upper row); (D) 126 kPa 0, 10, 20, 30, 40 and 50 min; (E) 740 kPa 0, 10, 20, 30, 40 and 50 min (lower row). *P-*values (*p*≤0.05) are indicated by an asterisk*.
(TIF)

S2 Fig Vascular index assessment using CD31 immunohistochemistry. Quantified CD31 labeling at 24 hours in mice bearing human PC3 xenografts treated with different ultrasound pressure and hyperthermia duration: (A) 0 kPa 20 and 30 min; (B) 246 kPa 20 and 30 min; (C) 570 kPa 20 and 30 min (upper row); (D) 126 kPa 0, 10, 20, 30, 40 and 50 min; (E) 740 kPa 0, 10, 20, 30, 40 and 50 min (lower row). *P-*values (*p*≤0.05) are indicated by an asterisk*.
(TIF)

S3 Fig Quantification of untreated tumor sections stained with (A) TUNEL (B) anti-CD31 antibody (C) Masson's trichrome and (D) Ki-67 staining at week 4.
(TIF)

## References

[1] van der Zee J. Heating the patient: A promising approach? Annals of Oncology. 2002 10.1093/annonc/mdf280 12181239

[2] Diederich CJ. Thermal ablation and high-temperature thermal therapy: Overview of technology and clinical implementation. International Journal of Hyperthermia. 2005 pp. 745–753. 10.1080/02656730500271692 16338857

[3] Hildebrandt B, Wust P, Ahlers O, Dieing A, Sreenivasa G, Kerner T, et al The cellular and molecular basis of hyperthermia. Critical Reviews in Oncology/Hematology. 2002 10.1016/S1040-8428(01)00179-2 12098606

[4] Diederich CJ, Hynynen K. Ultrasound technology for hyperthermia. Ultrasound in Medicine and Biology. 1999 10.1016/S0301-5629(99)00048-4 10461714

[5] Wust P, Hildebrandt B, Sreenivasa G, Rau B, Gellermann J, Riess H, et al Hyperthermia in combined treatment of cancer. Lancet Oncology. 2002 10.1016/S1470-2045(02)00818-5 12147435

[6] Falk MH, Issels RD. Hyperthermia in oncology. Int J Hyperthermia. 2001; 10.1080/02656730150201552 11212876

[7] Pelz JOW, Vetterlein M, Grimmig T, Kerscher AG, Moll E, Lazariotou M, et al Hyperthermic intraperitoneal chemotherapy in patients with peritoneal carcinomatosis: role of heat shock proteins and dissecting effects of hyperthermia. Ann Surg Oncol. 2013; 10.1245/s10434-012-2784-6 23456378

[8] Chicheł A, Skowronek J, Kubaszewska M, Kanikowski M. Hyperthermia–description of a method and a review of clinical applications. Reports Pract Oncol Radiother. 2007; 10.1016/S1507-1367(10)60065-X

[9] Marmottant P, Hilgenfeldt S. Controlled vesicle deformation and lysis by single oscillating bubbles. Nature. 2003; 10.1038/nature01613 12736680

[10] Sirsi S, Feshitan J, Kwan J, Homma S, Borden M. Effect of microbubble size on fundamental mode high frequency ultrasound imaging in mice. Ultrasound Med Biol. 2010; 10.1016/j.ultrasmedbio.2010.03.015 20447755 PMC2878876

[11] McDannold N, Vykhodtseva N, Hynynen K. Blood-Brain Barrier Disruption Induced by Focused Ultrasound and Circulating Preformed Microbubbles Appears to Be Characterized by the Mechanical Index. Ultrasound Med Biol. 2008; 10.1016/j.ultrasmedbio.2007.10.016 18207311 PMC2442477

[12] Karshafian R, Giles A, Burns PN, Czarnota GJ. Ultrasound-activated microbubbles as novel enhancers of radiotherapy in leukemia cells in vitro. Proceedings—IEEE Ultrasonics Symposium. 2009. 10.1109/ULTSYM.2009.5441487

[13] Ghoshal G, Oelze ML. Enhancing cell kill in vitro from hyperthermia through pre-sensitizing with ultrasound-stimulated microbubbles. J Acoust Soc Am. 2015; 10.1121/1.4936644 26723356 PMC4670444

[14] Czarnota GJ, Karshafian R, Burns PN, Wong S, Al Mahrouki A, Lee JW, et al Tumor radiation response enhancement by acoustical stimulation of the vasculature. Proc Natl Acad Sci. 2012; 10.1073/pnas.1200053109 22778441 PMC3409730

[15] Tran WT, Iradji S, Sofroni E, Giles A, Eddy D, Czarnota GJ. Microbubble and ultrasound radioenhancement of bladder cancer. Br J Cancer. 2012; 10.1038/bjc.2012.279\nbjc2012279 [pii] PMC3405216 22790798

[16] Al-Mahrouki A a, Iradji S, Tran WT, Czarnota GJ. Cellular characterization of ultrasound-stimulated microbubble radiation enhancement in a prostate cancer xenograft model. Dis Model Mech. 2014; 10.1242/dmm.012922 24487407 PMC3944496

[17] Lai P, Tarapacki C, Tran WT, Kaffas A El, Hupple C, Iradji S, et al Breast tumor response to ultrasound mediated excitation of microbubbles and radiation therapy in vivo. Oncosciencencoscience. 2016; 98–108. 10.18632/oncoscience.299 PMC4872648 27226983

[18] Fajardo LF, Prionas SD, Kowalski J, Kwan HH. Hyperthermia Inhibits Angiogenesis. Radiat Res. 1988; 10.2307/3577226 2453896

[19] Norton KA, Popel AS. Effects of endothelial cell proliferation and migration rates in a computational model of sprouting angiogenesis. Sci Rep. 2016; 10.1038/srep36992 27841344 PMC5107954

[20] Leung DW, Cachianes G, Kuang WJ, Goeddel D V., Ferrara N. Vascular endothelial growth factor is a secreted angiogenic mitogen. Science (80-). 1989; 10.1126/science.2479986 2479986

[21] Ferrara N, Houck K, Jakeman L, Leung DW. Molecular and biological properties of the vascular endothelial growth factor family of proteins. Endocr Rev. 1992; 10.1210/edrv-13-1-18 1372863

[22] Connolly DT, Olander J V., Heuvelman D, Nelson R, Monsell R, Siegel N, et al Human vascular permeability factor. Isolation from U937 cells. J Biol Chem. 1989; 2584205

[23] Sawaji Y, Sato T, Takeuchi A, Hirata M, Ito A. Anti-angiogenic action of hyperthermia by suppressing gene expression and production of tumour-derived vascular endothelial growth factor in vivo and in vitro. Br J Cancer. 2002; 10.1038/sj.bjc.6600268 12085210 PMC2746582

[24] Bauman TM, Nicholson TM, Abler LL, Eliceiri KW, Huang W, Vezina CM, et al Characterization of fibrillar collagens and extracellular matrix of glandular benign prostatic hyperplasia nodules. PLoS One. 2014; 10.1371/journal.pone.0109102 25275645 PMC4183548

[25] Al-Mahrouki AA, Karshafian R, Giles A, Czarnota GJ. Bioeffects of Ultrasound-Stimulated Microbubbles on Endothelial Cells: Gene Expression Changes Associated with Radiation Enhancement In Vitro. Ultrasound Med Biol. 2012; 10.1016/j.ultrasmedbio.2012.07.009 22980406

[26] Kim HC, Al-Mahrouki A, Gorjizadeh A, Karshafian R, Czarnota GJ. Effects of biophysical parameters in enhancing radiation responses of prostate tumors with ultrasound-stimulated microbubbles. Ultrasound Med Biol. 2013; 10.1016/j.ultrasmedbio.2013.01.012 23643061

[27] El Kaffas A, Al-Mahrouki A, Hashim A, Law N, Giles A, Czarnota GJ. Role of acid sphingomyelinase and ceramide in mechano-acoustic enhancement of tumor radiation responses. J Natl Cancer Inst. 2018;110: 1009–1018. 10.1093/jnci/djy011 29506145 PMC6136928

[28] Goertz DE, Todorova M, Mortazavi O, Agache V, Chen B, Karshafian R, et al Antitumor Effects of Combining Docetaxel (Taxotere) with the Antivascular Action of Ultrasound Stimulated Microbubbles. PLoS One. 2012; 10.1371/journal.pone.0052307 23284980 PMC3527530

[29] Todorova M, Agache V, Mortazavi O, Chen B, Karshafian R, Hynynen K, et al Antitumor effects of combining metronomic chemotherapy with the antivascular action of ultrasound stimulated microbubbles. Int J Cancer. 2013;132: 2956–2966. 10.1002/ijc.27977 23225339

[30] Kim BM, Hong Y, Lee S, Liu P, Lim JH, Lee YH, et al Therapeutic implications for overcoming radiation resistance in cancer therapy. International Journal of Molecular Sciences. 2015 10.3390/ijms161125991 26569225 PMC4661850

[31] Luqmani YA. Mechanisms of drug resistance in cancer chemotherapy. Medical Principles and Practice. 2005 10.1159/000086183 16103712

[32] Martin T, Ye L, Sanders A, Lane J, Jiang W. Cancer Invasion and Metastasis: Molecular and Cellular Perspective. Cancer Invasion and Metastasis: Molecular and Cellular Perspective. 2014.

[33] Weis SM, Cheresh DA. Tumor angiogenesis: Molecular pathways and therapeutic targets. Nature Medicine. 2011 10.1038/nm.2537 22064426

[34] Ziyad S, Iruela-Arispe ML. Molecular Mechanisms of Tumor Angiogenesis. Genes and Cancer. 2011; 10.1177/1947601911432334 22866200 PMC3411131

[35] Kim HC, Al-Mahrouki A, Gorjizadeh A, Karshafian R, Czarnota GJ. Effects of biophysical parameters in enhancing radiation responses of prostate tumors with ultrasound-stimulated microbubbles. Ultrasound Med Biol. 2013; 10.1016/j.ultrasmedbio.2013.01.012 23643061

[36] Lin FC, Hsu CH, Lin YY. Nano-therapeutic cancer immunotherapy using hyperthermia-induced heat shock proteins: Insights from mathematical modeling. Int J Nanomedicine. 2018; 10.2147/IJN.S166000 29950833 PMC6016258

[37] Haghniaz R, Umrani RD, Paknikar KM. Temperature-dependent and time-dependent effects of hyperthermia mediated by dextran-coated La0.7Sr0.3MnO3: In vitro studies. Int J Nanomedicine. 2015; 10.2147/IJN.S78167 25759583 PMC4346362

[38] Horsman MR, Overgaard J. Hyperthermia: a Potent Enhancer of Radiotherapy. Clin Oncol. 2007; 10.1016/j.clon.2007.03.015 17493790

[39] Garcia-Barros M, Paris F, Cordon-Cardo C, Lyden D, Rafii S, Haimovitz-Friedman A, et al Tumor response to radiotherapy regulated by endothelial cell apoptosis. Science (80-). 2003;300: 1155–1159. 10.1126/science.1082504 12750523

[40] Peñ LA, Fuks Z, Kolesnick RN. Radiation-induced Apoptosis of Endothelial Cells in the Murine Central Nervous System: Protection by Fibroblast Growth Factor and Sphingomyelinase Deficiency. CANCER Res. 2000; 10.1016/0360-3016(80)90175-3 10667583

[41] Paris F, Fuks Z, Kang A, Capodieci P, Juan G, Ehleiter D, et al Endothelial apoptosis as the primary lesion initiating intestinal radiation damage in mice. Science (80-). 2001;293: 293–297. 10.1126/science.1060191 11452123

[42] Roti JL. Cellular responses to hyperthermia (40–46°C): Cell killing and molecular events. International Journal of Hyperthermia. 2008 10.1080/02656730701769841 18214765

[43] Li L, Tan H, Gu Z, Liu Z, Geng Y, Liu Y, et al Heat stress induces apoptosis through a Ca2+-mediated mitochondrial apoptotic pathway in human umbilical vein endothelial cells. PLoS One. 2014; 10.1371/journal.pone.0111083 25549352 PMC4280109

[44] Song CW. Effect of local hyperthermia on blood flow and microenvironment: A review. Cancer Res. 1984; 6467226

[45] Song CW, Park HJ, Lee CK, Griffin R. Implications of increased tumor blood flow and oxygenation caused by mild temperature hyperthermia in tumor treatment. International Journal of Hyperthermia. 2005 10.1080/02656730500204487 16338859

[46] Zhang Y, Zhan X, Xiong J, Peng S, Huang W, Joshi R, et al Temperature-dependent cell death patterns induced by functionalized gold nanoparticle photothermal therapy in melanoma cells. Sci Rep. 2018; 10.1038/s41598-018-26978-1 29880902 PMC5992202

[47] Vorotnikova E, Ivkov R, Foreman A, Tries M, Braunhut SJ. The magnitude and time-dependence of the apoptotic response of normal and malignant cells subjected to ionizing radiation versus hyperthermia. Int J Radiat Biol. 2006; 10.1080/09553000600876678 16966182

[48] Baronzio GF, Hager ED, Baronzio GF, Delia Seta R, D’Amico M, Baronzio A, et al Effects of Local and Whole Body Hyperthermia on Immunity. Hyperthermia in Cancer Treatment: A Primer. 2006 10.1007/978-0-387-33441-7_20

[49] Kondo T, Matsuda T, Tashima M, Umehara H, Domae N, Yokoyama K, et al Suppression of heat shock protein-70 by ceramide in heat shock-induced HL-60 cell apoptosis. J Biol Chem. 2000; 10.1074/jbc.275.12.8872 10722733

[50] El Kaffas A, Nofiele J, Giles A, Cho S, Liu SK, Czarnota GJ. DLL4-notch signalling blockade synergizes combined ultrasound-stimulated microbubble and radiation therapy in human colon cancer xenografts. PLoS One. 2014; 10.1371/journal.pone.0093888 24736631 PMC3988033

[51] Mantso T, Vasileiadis S, Lampri E, Botaitis S, Perente S, Simopoulos C, et al Hyperthermia suppresses post—In vitro proliferation and tumor growth in murine malignant melanoma and colon carcinoma. Anticancer Res. 2019; 10.21873/anticanres.13347 31092422

[52] Zhang B, Zhou H, Cheng Q, Lei L, Hu B. Low-frequency low energy ultrasound combined with microbubbles induces distinct apoptosis of A7r5 cells. Mol Med Rep. 2014; 10.3892/mmr.2014.2654 25324182

[53] Li HX, Zheng JH, Ji L, Liu GY, Lv YK, Yang D, et al Effects of low-intensity ultrasound combined with low-dose carboplatin in an orthotopic hamster model of tongue cancer: A preclinical study. Oncol Rep. 2018; 10.3892/or.2018.6262 29436690 PMC5868397

[54] Nishimura Y, Urano M. Timing and sequence of hyperthermia in fractionated radiotherapy of a murine fibrosarcoma. Int J Radiat Oncol Biol Phys. 1993; 10.1016/0360-3016(93)90386-A 8226155

[55] Overgaard J. Influence of sequence and interval on the biological response to combined hyperthermia and radiation. Natl Cancer Inst Monogr. 1982; 7177183

[56] Li GC, Kal HB. Effect of hyperthermia on the radiation response of two mammalian cell lines. Eur J Cancer. 1977; 10.1016/0014-2964(77)90231-6 844475

[57] Dings RPM, Loren ML, Zhang Y, Mikkelson S, Mayo KH, Corry P, et al Tumour thermotolerance, a physiological phenomenon involving vessel normalisation. Int J Hyperth. 2011; 10.3109/02656736.2010.510495 21204622 PMC3086848

[58] Hegyi G, Szigeti GP, Szász A. Hyperthermia versus oncothermia: Cellular effects in complementary cancer therapy. Evidence-based Complementary and Alternative Medicine. 2013 10.1155/2013/672873 23662149 PMC3638606

[59] Andocs G, Rehman MU, Zhao Q-L, Tabuchi Y, Kanamori M, Kondo T. Comparison of biological effects of modulated electro-hyperthermia and conventional heat treatment in human lymphoma U937 cells. Cell Death Discov. 2016; 10.1038/cddiscovery.2016.39 27551529 PMC4979466

