# Supplementary material for: Ultrasound microbubble potentiated enhancement of hyperthermia-effect in tumours
Source: PLoS One. 2019 Dec 18;14(12):e0226475. doi: 10.1371/journal.pone.0226475 (PMC6919613; doi:10.1371/journal.pone.0226475)

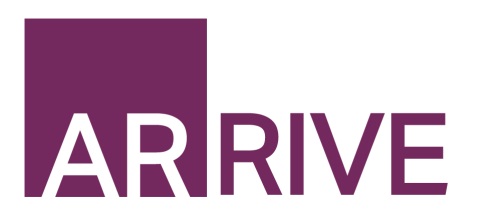


The ARRIVE Guidelines Checklist

Animal Research: Reporting In Vivo Experiments

Carol Kilkenny^1^, William J Browne^2^, Innes C Cuthill^3^, Michael Emerson^4^ and Douglas G Altman^5^

*^1^The National Centre for the Replacement, Refinement and Reduction of Animals in Research, London, UK, ^2^School of Veterinary Science, University of Bristol, Bristol, UK, ^3^School of Biological Sciences, University of Bristol, Bristol, UK, ^4^National Heart and Lung Institute, Imperial College London, UK, ^5^Centre for Statistics in Medicine, University of Oxford, Oxford, UK.*

|  | | ITEM | RECOMMENDATION | Section/ Paragraph |
| --- | --- | --- | --- | --- |
| 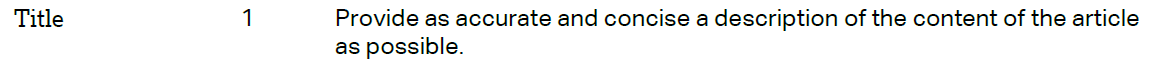 | | | Page 1 |  |
| 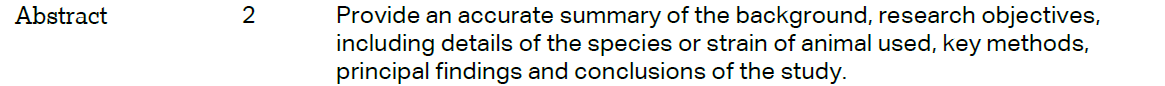 | | | Page 2-3 |  |
| INTRODUCTION | | |  |  |
| 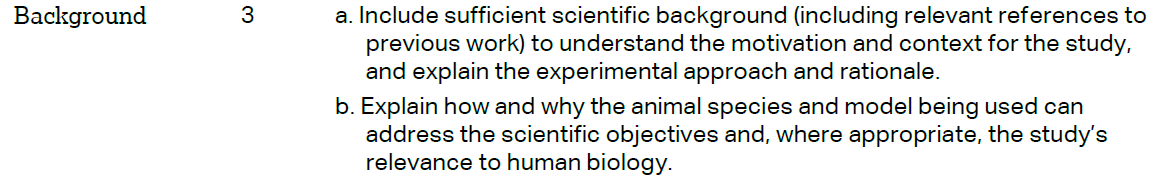 | | | Page 4-6 |  |
| 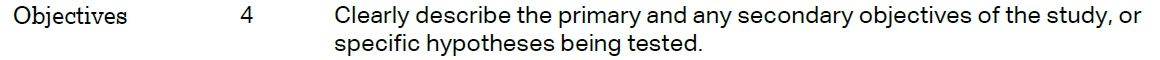 | | | Page 5-7 |  |
| METHODS | | |  |  |
| 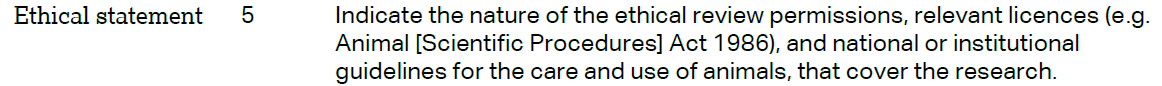 | | | Page 9 |  |
| 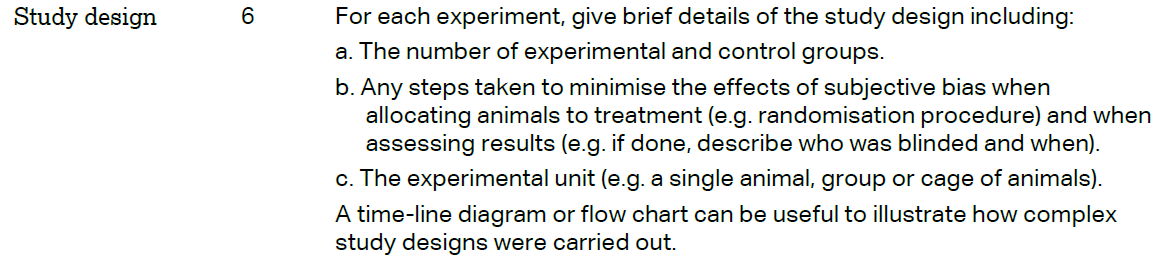 | | | Page 9-10 |  |
| 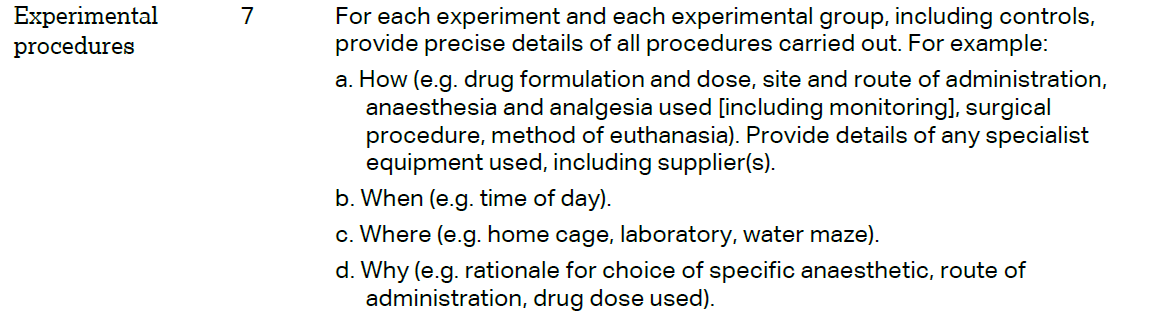 | | | Page 8-13 |  |
| 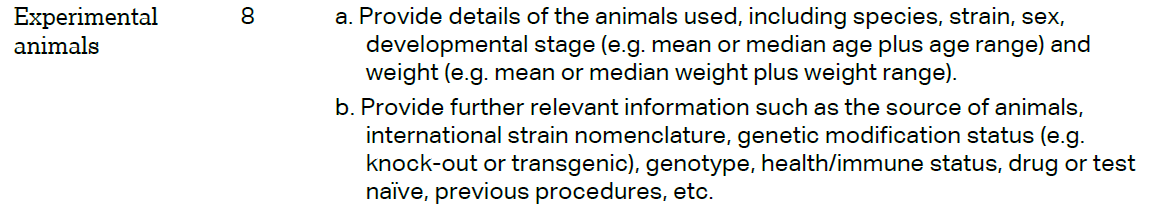 | | | Page 8 |  |

The ARRIVE guidelines. Originally published in *PLoS Biology*, June 2010^1^

| 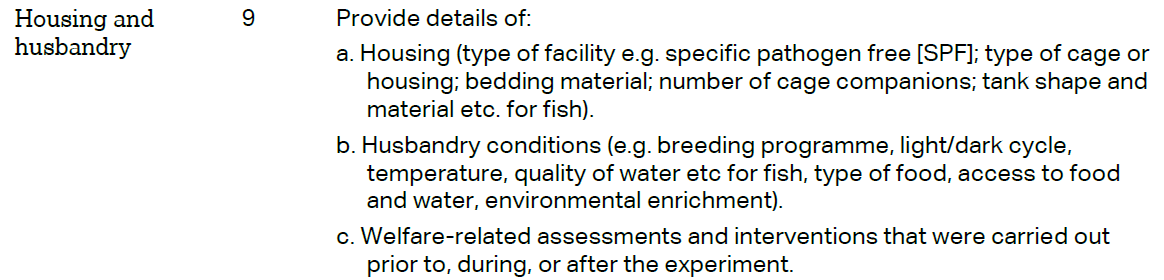 | a. SPF facility; 140 cage Allentown mouse vented rack, the cages are from Allentown and the version of the cages is Jag75; bedding material is corn cobs; singly housed animals to a max of 5 animals per cage. b. 12 hr. light/12 hr. dark cycle, the temperature is kept between 20-23 degrees C, automatic water is reverse osmosis with bleach. Water bottles provide autoclaved water. Animals always have as access to food and water, each cage has a plastic tube, plastic dome or paper house, also included crinkled paper and nestle for bedding material. c. All experimental mice were monitored daily for hydration, weight, and movement following the guidelines of the animal care committee at Sunnybrook Health Science Centre (Comparative Research). | |
| --- | --- | --- |
| 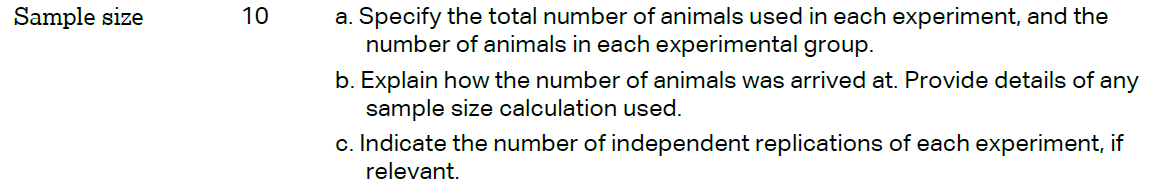 | Page 9-10 | |
| 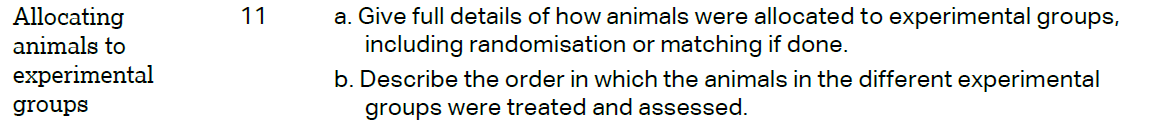 | Page 9-10 | |
| 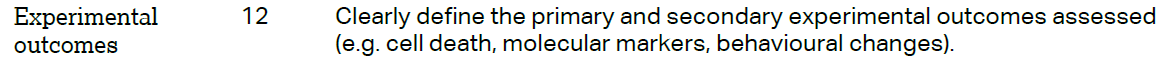 | Page 14-22 | |
| 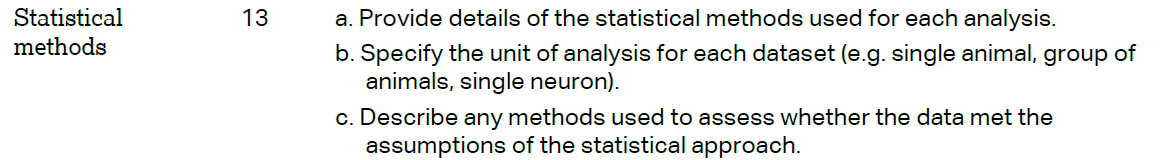 | Page 13-22 | |
| RESULTS |  | |
| 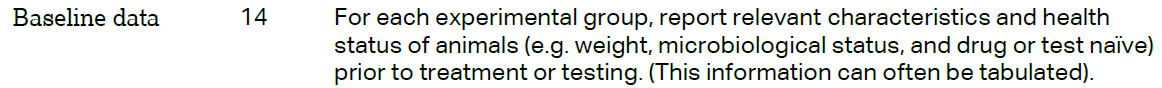 | All experimental mice were monitored daily for hydration, weight, and movement following the guidelines of the animal care committee at Sunnybrook Health Science Centre (Comparative Research). | |
| 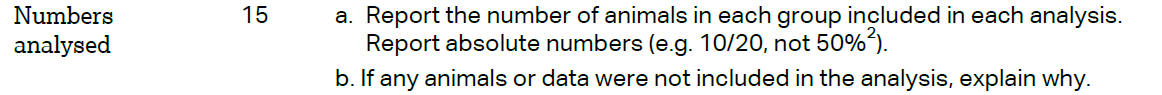 | Page 14 | |
| 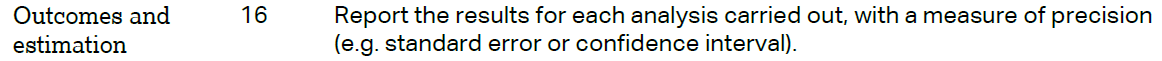 | Page 14-22 | |
| 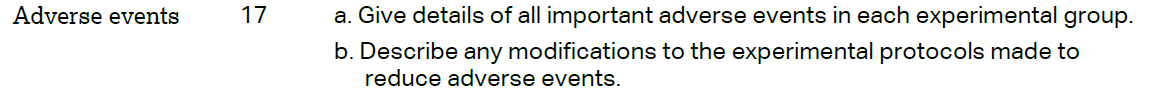 | No adverse events were observed during experiments. | |
| DISCUSSION |  | |
| 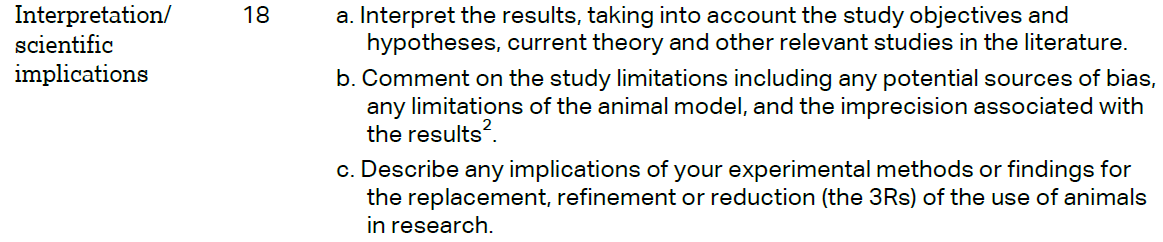 | Page 23-32 | |
| 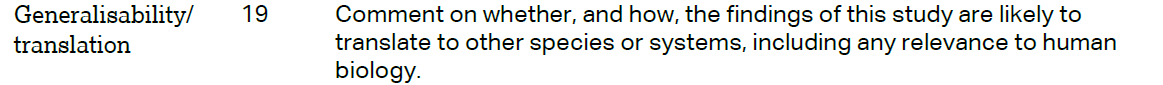 | Page 32 | |
| 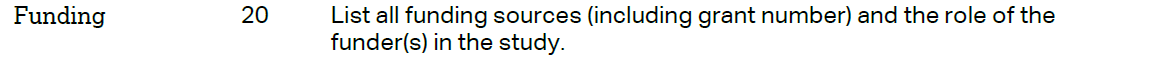 | | Page 9 |


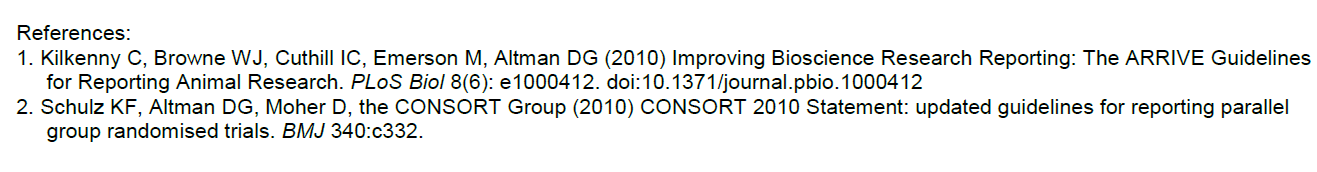

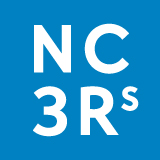

Supplement: S1 ARRIVE checklist — (DOCX) [file pone.0226475.s001.docx]
