# Supplementary figures and images for: Ultrasound microbubble potentiated enhancement of hyperthermia-effect in tumours
Source: PLoS One. 2019 Dec 18;14(12):e0226475. doi: 10.1371/journal.pone.0226475 (PMC6919613; doi:10.1371/journal.pone.0226475)

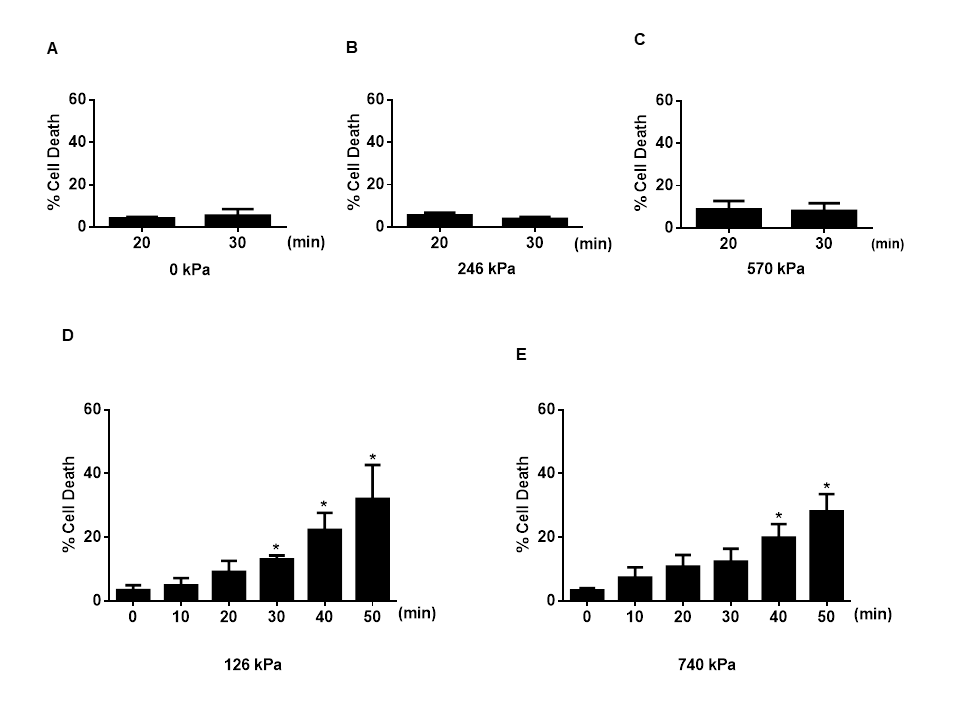

Supplement: S1 Fig — Quantified cell death staining at 24 hours following treatment of PC3 xenografts with varying ultrasound pressure and hyperthermia duration: (A) 0 kPa 20 and 30 min; (B) 246 kPa 20 and 30 min; (C) 570 kPa 20 and 30 min (upper row); (D) 126 kPa 0, 10, 20, 30, 40 and 50 min; (E) 740 kPa 0, 10, 20, 30, 40 and 50 min (lower row). P-values (p≤0.05) are indicated by an asterisk*. (TIF) [file pone.0226475.s002.tif]

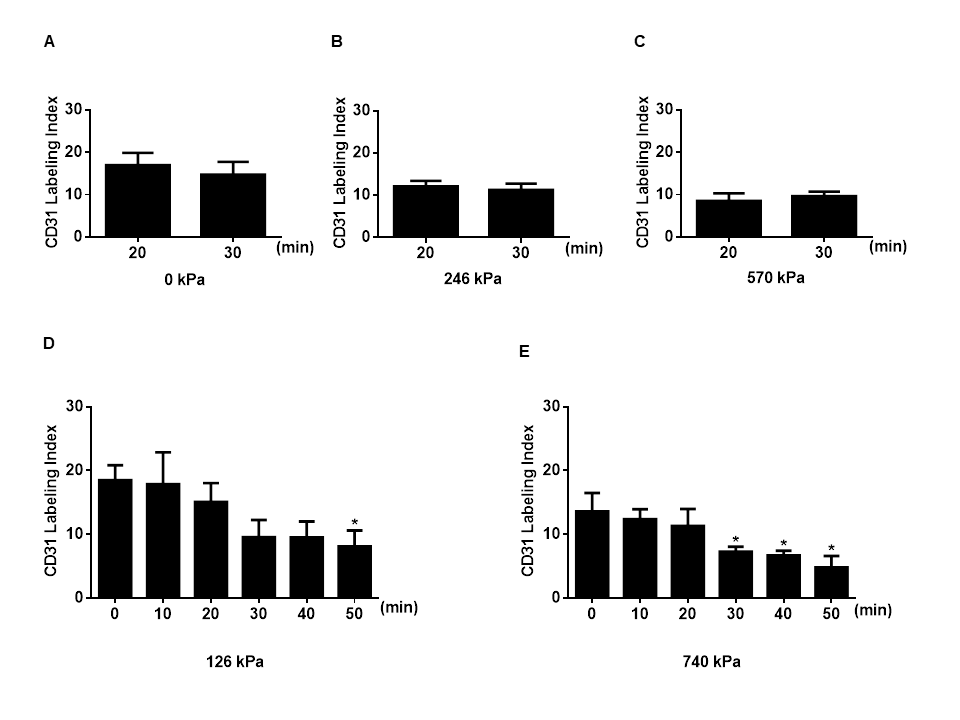

Supplement: S2 Fig — Quantified CD31 labeling at 24 hours in mice bearing human PC3 xenografts treated with different ultrasound pressure and hyperthermia duration: (A) 0 kPa 20 and 30 min; (B) 246 kPa 20 and 30 min; (C) 570 kPa 20 and 30 min (upper row); (D) 126 kPa 0, 10, 20, 30, 40 and 50 min; (E) 740 kPa 0, 10, 20, 30, 40 and 50 min (lower row). P-values (p≤0.05) are indicated by an asterisk*. (TIF) [file pone.0226475.s003.tif]

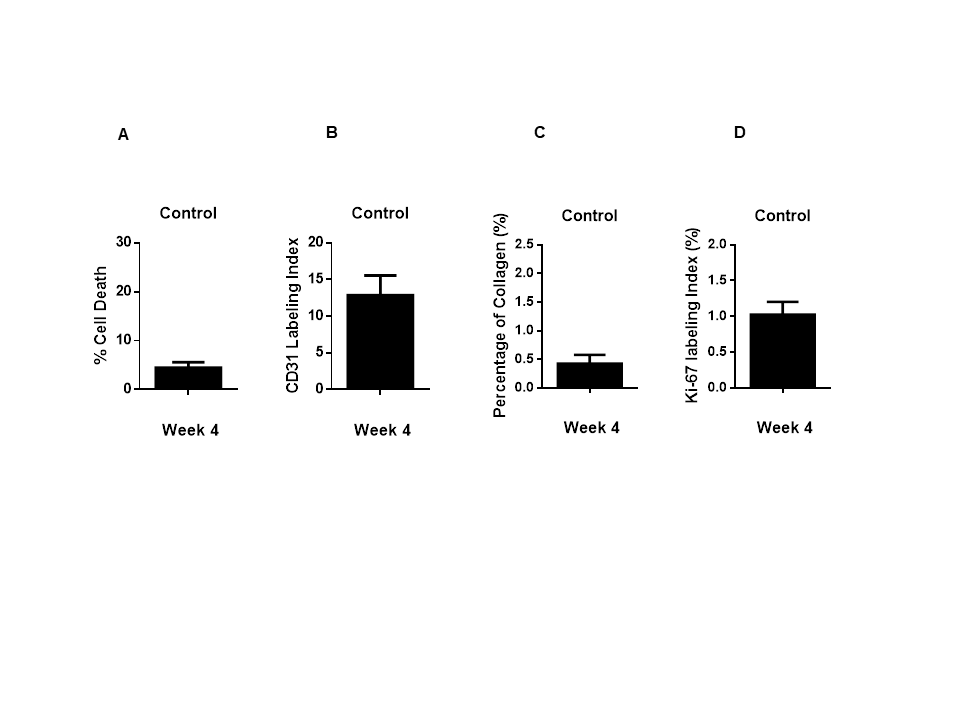

Supplement: S3 Fig — Quantification of untreated tumor sections stained with (A) TUNEL (B) anti-CD31 antibody (C) Masson's trichrome and (D) Ki-67 staining at week 4. (TIF) [file pone.0226475.s004.tif]
